# Artificial Intelligence (AI)-Based Systems Biology Approaches in Multi-Omics Data Analysis of Cancer

**DOI:** 10.3389/fonc.2020.588221

**Published:** 2020-10-14

**Authors:** Nupur Biswas, Saikat Chakrabarti

**Affiliations:** Structural Biology and Bioinformatics Division, CSIR-Indian Institute of Chemical Biology, IICB TRUE Campus, Kolkata, India

**Keywords:** artificial intelligence (AI), multi-omics analyses, cancer, machine learning, precision medicine

## Abstract

Cancer is the manifestation of abnormalities of different physiological processes involving genes, DNAs, RNAs, proteins, and other biomolecules whose profiles are reflected in different omics data types. As these bio-entities are very much correlated, integrative analysis of different types of omics data, multi-omics data, is required to understanding the disease from the tumorigenesis to the disease progression. Artificial intelligence (AI), specifically machine learning algorithms, has the ability to make decisive interpretation of “big”-sized complex data and, hence, appears as the most effective tool for the analysis and understanding of multi-omics data for patient-specific observations. In this review, we have discussed about the recent outcomes of employing AI in multi-omics data analysis of different types of cancer. Based on the research trends and significance in patient treatment, we have primarily focused on the AI-based analysis for determining cancer subtypes, disease prognosis, and therapeutic targets. We have also discussed about AI analysis of some non-canonical types of omics data as they have the capability of playing the determiner role in cancer patient care. Additionally, we have briefly discussed about the data repositories because of their pivotal role in multi-omics data storing, processing, and analysis.

## Introduction

Cancer is a complex heterogeneous disease (1). It is a consequence of malfunction and alteration of different biological entities, namely, genes, proteins, mRNAs, miRNAs, metabolites, etc., at a global scale. The human body contains almost ∼20,000 proteins (2), 20,000–22,000 protein-coding genes (3), ∼30,000 mRNAs (4), 2300 miRNAs (5), and 114,100 metabolites (6), respectively. Comprehensive analyses of these large numbers of bio-entities create several types of biological “omics” data. Cutting-edge technologies have made possible global profiling of a large number of genes (genomics and epigenomics), proteins (proteomics and phospho-proteomics), RNAs (RNA transcriptomics), miRNAs (miRNA transcriptomics), and metabolites (metabolomics) from the same individuals. Classical pathological diagnosis, which includes histopathology images and several types of blood tests, are essential for primary diagnosis and defining cancer stages. However, the pathological data has limitation of inferring any molecular basis of the disease. On the other hand, analysis of any single type of omics data is mostly limited to identifying the variation or at most correlation between one or two types of bio-entities. Its outcome is limited to reactive processes rather than causative phenomena (7). However, the aforementioned bio-entities are very much interrelated, acknowledging the central dogma of molecular biology (8). For example, an upregulated mRNA may or may not enhance its target protein expression. This protein, if it is an enzyme, will influence its associated metabolites. miRNAs also play a major role in this kind of scenario due to their inhibitory role by silencing or degrading mRNAs. So, the regulatory mechanisms are distributed across different types of bio-entities or, in another way, in different layers. Hence, studying only mRNAs, miRNAs, or proteins is not sufficient to understanding the complex disease etiology in which multiple bio-entities become dysfunctional. Interconnectivity and interdependence of various bio-entities demand a holistic approach utilizing a far more exhaustive and comprehensive application and integration of multi-omics data. Multi-omics analysis provides the path of information flow from one omics data to other omics data (9). Because of the involvement of a large number of entities, these omics data appear as “big” data in the biological context. The heterogeneous nature of cancer makes this data highly divergent from patient to patient. It demands profiling of multi-omics data at an individual level, subsequent analysis, and interpretation for the understanding of underlying biological phenomena and leads to the development of the field of “precision medicine” (10–12).

The aforementioned omics data can be considered as “primary” types of omics data as they are the direct outcome of several bio-entities. Apart from these “primary” types of omics data, there exist few other omics data, which are of non-canonical types, such as immunomics, microbiome data, and multiplex family history data, which belong to this category. These non-canonical data are integration of primary omics data and other non-omics biological information. This integration is a challenging work because of the heterogeneity, size, and complex relationship between the data (13). There is immense scope of using AI to build constructive models for analysis of non-canonical data.

Multiple databases like TCGA (14) and ICGA (15) are growing fast to accommodate multi-omics data. Rapid analysis of this massive amount of data is beyond human capability. Small sample size and large dimension of omics data limit the applicability of many conventional statistical methods. On the other hand, artificial intelligence (AI), a rapidly evolving branch of computer science, offers advanced analytical methods with predictive capabilities. The analysis and interpretation of multi-omics data demand the successful collaboration of biologists with computer scientists. Machine learning (ML) is a branch of AI. ML deals with computer programs where programs learn automatically from their earlier experience. The program initially performs some tasks, measures performance, gains experience, and then learns from experience; it performs remaining tasks to provide better performance (16). ML algorithms are initially trained using almost 70–80% of the whole data set, and the remaining 30–20% of data is used to validate the model followed by the algorithm. Then, the “trained” model is used to perform on new data. As ML algorithms learn from their experience, depending on the types of feedbacks available from earlier experiences, there are three types of learning, unsupervised, reinforcement, and supervised learning (17). In unsupervised or descriptive learning, the program learns patterns in input data without any explicit feedback from the learning. The usual goal is to find interesting patterns in the data without any labeled examples or prior information of the desired output for each input. As unsupervised learning does not require any manual effort for labeling the data, it is more widely applicable to address problems aimed to find clusters (18). On the other hand, in case of supervised learning, the program learns the mapping of input data in output from some example labeled set of input–output pairs. Depending on the nature of the output variables, supervised learning algorithms deal with two types of problems. The problem is called classification if the output is categorical, and the problem is called regression when the output is real-valued (18). There is another type of learning called reinforcement learning, which is in between supervised and unsupervised learning because here the program learns from reinforcement (17). In this case, the program learns from the gain or loss in the output result through trial-and-error interactions with a dynamic environment (19, 20). Among these three categories, reinforcement learning is relatively less used for multi-omics data analysis. Developing the methodologies is an active area of research (21–25). Pan-cancer analysis is also being done. Broadly, there are three types of integrations of multi-omics data, namely, model based, concatenation based, and transformation based (26).

**FIGURE 1:**
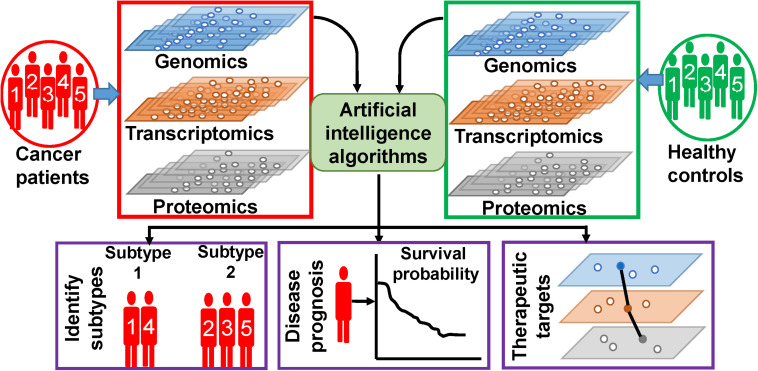
Artificial intelligence (AI)-based analysis of multi-omics data. Different types of omics data are integrated and analyzed by AI algorithms to extract patient-specific information.

In this review, we are trying to discuss the methodologies and outcomes of AI on the analysis of multi-omics data, specific to cancers. The following section discusses about few methodologies of ML algorithms, which are frequently used for multi-omics data analysis. Based on our observations on the ongoing research works, this review is broadly concentrated on three types of works, which is illustrated in Figure 1. First, we will discuss on findings of AI on the classification of healthy controls and cancer patients as well as on finding out subtypes of different types of cancer. Second, we will emphasize on outcomes of AI on cancer prognosis. Third, we will present the AI-based efforts on identification of novel therapeutic targets. In the fourth section, we discuss about few other types of associated data whose inclusion is expected to give more fruitful outcomes. In this section, we have also discussed about data repositories. In the fifth section, we briefly discuss about precision medicine approaches. Finally, we discuss the future challenges and provide our conclusions. We have also enlisted and summarized the major techniques and algorithms used for this purpose along with their specialties and outcomes in Table 1. However, in this review, we have excluded the impact and analysis of radiomics data. Radiomics data deals with the analysis of different types of radiological images of tumor sites, and hence, it is not a direct outcome of the aforementioned bio-entities, although the inclusion of radiomics with the multi-omics analysis is going to be a powerful tool for identifying distinct cellular subtypes in a given type of cancer (27, 28). Radiomics analyses utilize various kinds of diagnostic image data for better prediction of cancer diagnostics and prognostics, clinical outcome, and survival. Image analysis, ML, and AI have been successfully used in radiomics analysis. Hence, we believe that the nature of radiomics analysis including image analysis, image processing, types of images, and integration of image analysis along with molecular and clinical data demand an extended review work separately.

**TABLE 1:** Features of recent methodologies and techniques used for AI based systems biology approaches in multi-omics data analysis of cancer.

Methodology	Techniques	Characteristics	Specialty	Cancer types	Omics data	Outcome	Performance	References
Unsupervised and supervised	Stacked autoencoder and hierarchical integration deep flexible neural forest network (HI-DFN Forest)	Autoencoders are used to integrate multi-omics data. HI-DFN Forest is used for classification	Considers intrinsic statistical properties and learns high-level representations of each omics data. HI-DFNForest model is suitable for small-scale data.	Breast, glioblastoma, ovarian cancer	mRNA expression, miRNA expression, methylation	Classify cancer subtypes	Accuracy: 0.885 (glioblastoma multiforme)	(33)
Supervised and unsupervised	Deep-learning, autoencoder	Autoencoder was used to reduce data and then SVM to find sub-groups.	Predicts survival subgroups and aggregates genes belonging to similar pathways.	Hepatocellular carcinoma	mRNA expression, miRNA expression, methylation	Predict survival subgroups	Concordance index: 0.68	(34, 35)
Unsupervised	Combinations of autoencoders	Data integration based on four types of variational autoencoders (VAE)	All VAE architectures perform well. Learned representations coupled with SVMs provides best prediction.	Breast cancer	mRNA expression, CNV data	Focused on data integration approaches	Accuracy: 0.858	(37)
Kernel framework	Multiple kernel learning	Combine several kernels to one meta-kernel in an unsupervised framework.	Identifies cancer subtypes and provides relationships between them	Breast cancer	mRNA expression, miRNA expression, methylation	Proposed generic approach of data integration	Average cluster purity: 0.70	(39)
Unsupervised	Autoencoder	multi-modal sparse denoising autoencoder framework coupled with sparse non-negative matrix factorization	Illustrate impact of individual omics feature on pathway score.	Colorectal cancer, lung squamous cell carcinoma, glioblastoma multiforme and breast cancer	mRNA expression, miRNA expression, methylation, CNV	Cluster patients and provide feature pathways for patient clusters	Consensus silhouette index: 0.98 (colorectal cancer)	(42)
Supervised and unsupervised	Random forest, SCVM	Combined use of random forest and SVM	Classifies normal and cancer samples across different tissue types and hence useful for diagnosis	9 types of cancers	Pan-cancer mRNA expression,	Classification and identifies biomarkers	Accuracy: 97.89% (non-specific tissue type)	(43)
Unsupervised	Autoencoder	Three types of integration approaches used. Feature combinations with highest average predictive accuracy was used.	Auto-encoder based classification	Neuroblastoma	mRNA expression, CNV	Prognosis sub-group	*p*-value from Kaplan-Meier curves for overall survival: 2.8e-8	(44)
Supervised and unsupervised	Integrative network fusion network and deep learning	Random forest was trained by two types of integrated omics data. Classifier was used based on intersection of two training processes.	Two approaches followed for data integration, juxtaposed and integration by similarity network fusion.	Neuroblastoma	mRNA expression, CNV	Prognosis sub-group	*p*-value for Kaplan-Meier plot: 5.7e-4	(45)
Supervised and unsupervised	SVM, and random forest	Initial supervised analysis was followed by systems biology approach and random forest based analysis	Multi-omics data was integrated in multiple steps with removal of redundant features.	Colorectal cancer	mRNA expression, miRNA expression, CNV, metabolomics	Identifies markers, pathways associated with cancer relapse	*p*-value from Kaplan-Meier curves for overall survival: 5.7e-4	(46)
Multi-view learning	Min-Redundancy and Max-Relevance (MRMR)	Finds features having maximum relevance in feature selection and minimum redundancy with already selected features	Two stage feature selection framework	Ovarian cancer	mRNA expression, methylation, CNV	Identifies biomarkers for predicting survival.	Area under curve (AUC): 0.7 for random forest classifier	(47)
Neural network	Deep learning based neural network	Instead of gene expression data, eigengene modules of gene co-expression analysis were used as features.	Associates feature genes with metadata like age	Breast cancer	mRNA expression, miRNA expression, methylation, CNV and other metadata	Survival prediction	Mean concordance index: 0.6813	(48)
LASSO and neural network	Deep learning framework and lasso	Use group LASSO and deep neural network for data integration and then Cox model for survival prediction	Different features from same gene are grouped together	Pan-cancer	mRNA expression, CNV, SNP	Survival prediction	Concordance index: 0.8	(49)
Kernel method	Kernel alignment assessment of omic similarity matrix	Omic similarity matrix was constructed for each omics data and similarity between them was measured.	Considers involvement of large number of biomarkers in disease prognosis	Pan-cancer	mRNA expression, miRNA expression, methylation, CNV, SNP	Variation in prognosis assessment across cancer types	Concordance index >0.68 (sample size = 900)	(50)
Kernel based and feature-selection based	Bayesian efficient multiple kernel learning (BEMKL) model	Kernalized regression which works on similarities between cell lines	Reduces number of model parameters to match number of samples, not feature numbers. Extract non-linear relations between features and drug response.	Breast cancer cell lines	mRNA expression, CNV, methylation, SNP, proteomic	Drug-response prediction	False discovery rate: 2.5e-5	(51, 52, 57)
Deep neural network, transfer learning	Multi-Omics Late Integration (MOLI)	Creates feature space for each omics data. Learned features are integrated by concatenation and used for prediction of drug response. Use transfer learning by using responses of all drugs for same target while training.	Considers unique distribution for each omics data.	Pan-cancer	mRNA expression, CNV, SNP	Predicts drug response	Accuracy: 0.8 for drug cetuximab	(53)
Supervised	SVM and leave-one-out cross-validation (LOOCV)	Finds features from each omics data and then identifies marker candidates based on miRNA and mRNA interactions	Analyzed integrated mRNA and miRNA expression data considering their interactions	Pancreatic ductal carcinoma	MRNA expression, miRNA expression	Identify mRNA and miRNA markers. Predicts miRNA expression level	AUC: 0.925 for miR-21 as multi-marker	(54)
Supervised	idTRAX	Finds target kinases from the compound data of all genes	Identifies kinases as effective targets of drugs	Breast cancer	Genomic and transcriptomic	Cell-model selective anti-cancer drug target	Spearman correlation ∼0.1	(55)
Supervised	Capsule network based modeling (CapsNetMMD)	Multi-omics data is integrated to form feature matrix and converted to capsule layers by convolution.	Supervised classification is done based on known breast cancer genes	Breast cancer	mRNA expression, methylation, CNV	Therapeutic target genes of breast cancer	*p*-value: 3.6e-141 (rank cut-off: 20%)	(56)
Supervised	Random forest and different classifiers	Features were extracted based on shrunken centroid and random forest based algorithm. Different classifiers were used.	Considers methylation patterns. Distinguishes early and late stages of cancer.	Papillary renal cell carcinoma	mRNA expression, methylation	Finds driver genes	Accuracy: 84.6% for random forest	(58)
Semi-supervised	PLATYPUS	After training on labeled data, it co-trains with unlabeled data considering the messing data.	Important features are linked to drug sensitivity	Pan-cancer cell lines	mRNA expression, CNV, SNP	Predicts drug response	AUC: 0.9	(59)

## Major AI-Based Methodologies

The supervised learning-based support vector machine (SVM) algorithm is one of the widely used approaches for the analysis of multi-omics data. SVM creates a linear hyperplane, maintaining the largest possible distance between different classes of example data points. While being trained for a given task, ML algorithms also find out relevant features for better performance of a task. SVM-based methods are broadly used for finding subtypes in cancer as well as for extracting essential features (biomolecules) that play as a marker. The major goal of the classification is the reclassification of cancer based on molecular features rather than tissue type (29–31). Random forest (RF) algorithms are also frequently used. As the name suggests, the RF algorithm is composed of many decision trees. Each tree is grown using a training set and a random vector and works as a classifier. Each tree votes for the most popular class, and the most voted class is chosen (32). Apart from supervised learning-based SVM and RF algorithms, unsupervised learning methods like autoencoders are also used to reduce the “big” size of multi-omics data. Autoencoders consist of an encoder and a decoder. The encoder extracts features from large input data, and the decoder tries to construct an output very similar to the input using only the extracted features. In this way, it excludes the redundant data (18).

## AI in Cancer Classification and Subtype Determination

The outcome of treatments to cancer patients having similar pathological features differs greatly. For providing better treatment, patients having similar symptoms need to be further categorized. This categorization could be related to the nature and abundance of bio-entities for individual patients. With the use of AI, researchers have tried to find subtypes in different types of cancers based on the cluster of different genes, mRNAs, and miRNAs. The advantage of multi-omics integration over single omics data in the context of cancer subtype determination was illustrated using mRNA expression, miRNA expression, and DNA methylation data for three types of cancers, namely, breast cancer, glioblastoma, and ovarian cancer. The stacked autoencoder was used to each omics data. The extracted representations were integrated in another autoencoder. Finally, the complex representation was used in the deep flexible neural forest network model for subclassification of cancers (33). Application of supervised and unsupervised learning on RNA transcriptomics, miRNA transcriptomics, and DNA methylation data of hepatocellular carcinoma (HCC) has identified two subgroups of patients with significant survival differences (34). Extending this study to multiple types of cohorts of varying ethnicity have identified 10 consensus driver genes, significantly associated with patients’ survival (35). It also shows consensus driver mutations, and their copy-number variations are associated mostly with mRNA transcriptome and less with miRNA trasncriptome (35). ML-based multi-omics analysis has been applied to identify probable breast cancer patients. Analysis of proteomics and metabolomics data for 24 breast cancer patients and 61 healthy persons has categorized a healthy group of people into two subcategories of low-risk and high-risk (36). Different combinations of auto-encoders have been used to study the most effective approach of multi-omics data integration in the context of breast cancer (37). The multiple-kernel framework is also used to integrate multi-omics datasets and to find closeness between the subtypes of breast cancer. Kernels are ML methods where a function called kernel function maps non-linear data sets into a higher-dimensional space to make the data linearly separable and hence can be classified (38). By linearly combining multiple kernels, different breast cancer subtypes such as, Basal, LumA, LumB, and Her2 were differentiated along with their relations (39). Long non-coding RNAs were also identified, many of which were earlier not known in cancer. The expression of these RNAs determines survival probability (40). Machine learning-based multi-omics analysis of pan-cancer data shows the existence of clusters within different types of cancers (41). Using “feature” genes, patient clusters were further correlated with “feature” pathways. Using autoencoders, PathME provides patient-specific pathway scores for disease subtype identification (42). Employing the supervised learning algorithm and RF over nine tissue types, Mohammed et al. have shown successful classification between normal and cancer tissues when tissue type is specified as well as non-specified. They further identified genes as potential biomarkers and critical pathways for different tissue types (43).

## AI in Cancer Prognosis

Early detection and prediction of prognosis are essential requirements for limiting tumor growth by providing proper clinical care to cancer patients. As disease prognosis differs across patients having the same cancer, AI has been used to find subcohorts within the patient cohorts based on the prognosis and survival data. Apart from finding subtypes, AI has identified biomarkers, which determine recurrence of cancer. AI has been applied to determine prognosis in high-risk neuroblastoma patients. Using overlapped gene expression and copy number alteration, data in unsupervised learning algorithm autoencoder identified relevant features which were used for clustering into two subgroups (44). In another study, as a part of neuroblastoma data integration challenge, Francescatto et al. have used the integrative network fusion framework along with the ML classifier to extract features which can discriminate between different outcomes of patients (45). In the case of colorectal cancer, cancer is prone to relapse for 20% of patients who were cured by surgery. One study has been conducted to obtain biomarkers for relapse. Using gene expressions, miRNA expressions, copy number variation data, and metabolomics data in a rigorous cross-validation approach of SVM and recursive feature elimination combined with random forest-based integrative analysis (RF-ACE) have identified markers for each type of data separately (46). Apart from supervised SVM, researchers have used the minimum redundancy and maximum relevance (MRMR) method to extract significant features for predicting the survival of ovarian cancer patients. The MRMR method iteratively selects multi-omics-derived features, which are maximally relevant for survival prediction and minimally redundant with the existing set of features (47). Deep learning-based neural networks also found its applicability in breast cancer survival prognosis. To avoid overfitting effects because of the large dimensionality of omics data, survival analysis algorithm SALMON works on eigengene matrices of co-expression network modules. To increase robustness, it integrates classic cancer biomarkers along with multi-omics data and identifies important feature genes and cytobands (48). Survival analysis is done using the deep learning framework with the hazard model and lasso regularization model for different types of cancer. The lasso model keeps only the relevant features. Information from the same gene across different types of omics data is grouped together and used for deep learning-based analysis which performed better (49). The kernel-based ML method quantified prognostic values of genomic, epigenomic, and transcriptomic data for 14 cancer types. The omics similarity matrix was constructed for each omics data using the kernel functions. Analysis over 3382 samples showed that the result is very much dependent on cancer type. For example, mRNA transcriptomic data shows the best prognostic value in lower-grade glioma. Inclusion of clinical variables provides significantly better prognostic value (50).

## AI in Identification of Therapeutic Targets

One of the basic requirements of precision oncology is predicting drug responses for a patient cohort. The benefits of ML methods have been tested for drug response modeling and prediction following both kernel-based and feature selection-based approaches (51). In a competitive challenge, DREAM7, responses of 28 drugs in growth inhibition of 53 breast cancer cell lines were ranked using different algorithms. Among them, the Bayesian multitask multiple-kernel learning method performed best (52). Deep neural network-based analysis has been employed for drug response prediction. MOLI, a multi-omics late integration method based on deep neural network, integrates somatic mutation, copy number aberration, and gene expression data to predict drug response behavior. In this method, features are extracted from different omics data separately and then integrated and optimized to train for predicting response of a specific drug. MOLI is also used for pan-drug data, data on drugs with the same target (53). SVM and leave-one-out cross-validation (LOOCV) model have been used to predict important features in RNA and miRNA transcriptomics data for 104 pancreatic ductal adenocarcinoma tissues and 17 normal tissues. These features (selected RNAs and miRNAs) combined with miRNA target expression data were further used to identify effective diagnostic markers which were validated in other independent datasets and biologically interpreted by pathway analysis of the corresponding target genes (54). Machine learning-based analysis has also been applied to identify cell-model-selective anticancer drug targets for breast cancer (55). The feature genes extracted from multi-omics data of breast cancer by capsule network-based modeling were compared with known cancer genes, and novel genes were extracted (56). Pan-cancer analysis of nine cancers has revealed that proteomics data combined with gene expression, miRNA expressions, and genomics performs better in predicting the sensitivity of chemotherapeutics and molecularly targeted compounds. This study was conducted over 58 cell lines across nine cancers using the Bayesian Efficient Multiple Kernel Learning (BEMKL) model (57). It validates the superiority of multi-omics data analysis across cancer types. Correlating methylated genes with their expression data in papillary renal cell carcinoma shows that hypomethylated genes are associated with immune function. Several tumor-suppressor genes appeared hypermethylated. Differentially methylated genes distinguished normal and cancer samples but failed to distinguish tumor samples based on the tumor stages. Feature selection methods based on RF and other methods were used to extract marker genes as well as to distinguish early and late stages of cancer (58). ML framework PLATYPUS extracts most informative features from different omics data and allows these features to vote on predicted patient outcome on drug responses. Many of these features are well-known targets of the drugs whose response were predicted (59).

## AI and Secondary Omics Data

Apart from primary types of omics data, which include transcriptomics, genomics, proteomics, and metabolomics, few other types of data are also becoming important. Here we will briefly discuss the role of immunomics, microbiome data, multilayer signature biomarkers, and multiplex family history data along with different data repositories.

### Immunomics Data

Immunomics and/or immune profiling provide “omics” information with various immune cellular types abundant at a given physiological context. Immunome refers to the set of genes and proteins that constitute the immune system. Immunomics or system immunology integrates different multi-omics data (e.g., genomics and proteomics) and clinical data with immunology to view the network of immunome and to understand the immune function at both single-cell level and population level (60–63). The immune cells (T cells, B cells, etc.) behave like ML algorithms. They can identify the antigens depending on their prior learning (64). As the T cell receptor (TCR) proteins present in the T cells determine the binding of antigens with the T cells, immuno-sequencing of TCRs determines the role of T cells in disease progression. AI can help to translate TCR sequences to antigens they can recognize. Companies like Microsoft are focused to get antigen-specific binding data for several diseases along with ovarian and pancreatic cancers (65). ML algorithms have an immense scope of application in immune-oncology, specifically in pattern recognition in histopathological images and in survival analysis (66). The immune response to cancer cells varies widely among people. The web server EpiToolKit offers a platform of different prediction methods for peptide–major histocompatibility complex (MHC) binding. These prediction methods often use AI (67). Single-cell expression profiles of tumor cells and immune cells identify genes and proteins associated with the tumor-specific immune system and can be targeted in immune therapy. The expression fold change of enriched genes and proteins predicts disease prognosis and determines treatment (68, 69). The multi-omics profile of the tumor microenvironment (TME) provides insights on intra-tumor heterogeneity paving the path of precision immune-oncology (70).

It is now widely established that tumor growth and dissemination result from a cross talk between cancer-cell-intrinsic factors and the immune system (71, 72). Studies have shown that the tumor-infiltrating immune cells of both myeloid and lymphoid origin exert a dynamic relationship with the tumor and have a significant impact on the clinical course of the disease (73). Compelling studies have pointed out the fact that improved survival of patients with ovarian cancer positively correlates with the abundance of T cells into the tumor site (74, 75). It appears that discrete TMEs with disparate immune parameters could be the underlying cause of differential prognosis of the disease. Therefore, deeper analysis of the complexity within the TME is important to gain an insight into the immune landscape, which could predict the responsiveness to the immunotherapeutic interventions. Moreover, it is also important to understand the intricate mechanism(s) leading to the dysfunctionality of T cells at the tumor site and identify the potential approach to reinvigorate their effector functions. Hence, comprehensive characterization of the immune cells present at the tumor sites followed by subsequent stratification of the abundance of immunosuppressive population patients can be largely benefited by AI-based models, which could aim to predict emergent immune signatures within the cancer patients.

### Microbiome Data

The human body acts as the host of swarm of microorganisms. These microorganisms play a symbiotic role in the well-being and their unbalance or dysbiosis is correlated with many diseases, including cancer. Inclusion of microbiome data with multi-omics data by several computational approaches has exemplified our understanding of complex host–microbiome interactions leading to microbiome-targeted drug discovery (76). The launch of the integrative Human Microbiome Project (iHMP) is a leveraging step toward that direction (77). As host–microbiome interactions include exchange of different small molecules, specifically metabolites and signaling molecules, metabolomics data appear as most informative (78). Databases are launched to share information on gut microbes paired with genome sequences and longitudinal multi-omics data (79). The intersection of ML with network biology will enrich microbiome research where many microorganisms still remain understudied (80, 81). Transfer learning, a branch of ML, provides opportunity to transfer the learned information from a well-studied species to an understudied species (82). Shotgun metagenomics data provide quantitative data and have been used for disease prediction for colorectal cancer across multiple cohorts (83). It is reported that the microbiome of breast tissue differs from that of skin tissue in case of breast cancer patients. The RF algorithm was able to predict tissue type based on microbiota profile (84). Random forest along with Bayes net algorithm performs well to predict colorectal cancer from fecal and gut microbiota (85). Cancer patients often die of bloodstream infections. ML has been used to predict risk of bloodstream infections from fecal microbiome data for patients before initiation of chemotherapy treatment (86).

### Multiplex Family History Data

Although genetic, in general cancer is not a hereditary disease. However, for some cancers a small fraction of cases appear familial (87). For example, hereditary non-polyposis colorectal cancer (HNPCC) contributes 5–10% of all colorectal cancers (CRC) (88, 89). It is also reported that families with history of nasopharyngeal carcinoma (NPC) have a greater risk of salivary, cervical, and gastric cancers in first-degree relatives of NPC patients (90). The inclusion of multiplex-family data from Taiwan shows co-aggregation of NPC within families (91). In a pan-cancer study, covering 25 most common cancers, the statistical analysis shows that relative risk of having cancer is high if a parent or sibling has concordant cancer, if multiple family members are affected. Also, the risk depends on the type of cancer (92). In this background, with the ease of multi-omics data profiling, multi-omics integrative analysis for such multiplex family can enrich our understanding of the underlying genetic architecture of cancer predisposition. To analyze this huge and complex interrelated data, ML algorithms are indispensable.

### Multilayer Signature Biomarkers

The comprehensive understanding of cancer progression leading to development novel diagnostic/prognostic markers and therapeutic interventions requires integration and utilization of diverse “omics” strategies at multiple levels. The underlined concept is that complex patho-physiological mechanisms can only be fully understood through the study of molecular interactions among different omics layers. Several studies have shown the importance of multidimensional approaches (such as genomics, transcriptomics, proteomics, metabolomics, immunomics, and metagenomics) to portray the complexity of cancer–host interactions. Recent technological advances have permitted high-throughput measurement of the human genome, epigenome, metabolome, transcriptome, and proteome at the population level. Each of these studies can offer complementary analyses of a certain biological function, and hence, integrative multi-omics analyses are needed to uncover synergistic interactions. However, because each omics study analyzes a different molecular layer, integrative analyses using different omics studies might have closely related biological functions and thus might directly interact at the network level. Therefore, it is possible to build network(s) with direct interactions among multiple molecular layers, characterized by higher network complexity. In addition, incorporating biological functionality from different molecular layers, such as RNA, proteome, and metabolome results, can boost the power of genetic mapping. Mathematical model-based system biology approaches are proven to be successful for signaling and metabolic network analysis. Mathematical models for signaling pathways have been developed based on logical models, kinetic models, decision tree, and differential equation-based models. Different omics data of metabolic gene expressions, protein levels, and metabolomes in different cancer are integrated to study metabolic regulation in cancer. However, development of integrative methods that aim to capture weak yet consistent patterns across data types which could be statistically associated with diagnostic and/or prognostics markers of the complex systemic diseases (e.g., cancer) is very limited.

A single platform for integrating and mining pan-omics entities derived from a large-scale cancer patient cohort and further analyzing them to derive meaningful cross correlation among multilayer data is due. Consistent patterns across data types could be statistically associated with diagnostic and/or prognostic markers of the complex systemic diseases like cancer. Hence, this kind of platforms and their derived results should aid researchers all over the country to identify novel biomarker signatures.

Metabolic reprogramming of tumor cells including their surrounding stromal environment may be mandatory in order for tumors to emerge and particularly to evolve into a more aggressive state. This metabolic switch has been entitled one of the new “hallmarks of cancer” (93), expanding the original set of hallmarks (94). Understanding the underlying mechanisms via integration of various cellular pathways is expected to help elucidate overall tumor pathogenesis. Despite the metabolic heterogeneity, certain metabolic patterns tend to be distinguishable in ovarian tumor in comparison to normal ovarian tissue. The question as to whether metabolic alterations in the TME merely represent by-products from oncogenesis and whether they function as “reactive” mediators of oncogenic process via altering the state of the tumor surrounding immune system sentinels will need to be further addressed in detail, in order to understand that one needs to first investigate the molecular mechanism by which the impact of signaling and transcriptional aberration is transgressed to metabolic reprogramming. To cope with the complexity of interconnected cellular pathways, efficient systems biological approaches need be developed. Further investigation is required to establish a link between distinct metabolomic outcome and immunological status of the cancer TME. Utilization of multi-omics data via mathematical modeling-based analysis in principle can identify cross-pathway links connecting signaling proteins or transcription factors or miRNAs to metabolic enzymes and their metabolites using network analysis and mathematical modeling. These types of cross pathway links were shown to play important roles in metabolic reprogramming in cancer scenarios such as glioblastoma multiforme (95). Therefore, integrative studies aided by multi-omics analysis, mathematical modeling, and deep learning-AI-based strategies would lead to the development of a more comprehensive understanding of cancer metastasis.

### Data and Model Resources

Multi-omics data is multi-dimensional in nature and “big” in size. Storage, hosting, and making the data accessible to researchers are also a challenging work. The data need to be stored anonymously maintaining quality. Omics data when publicly shared by the researcher provides scope for other researchers for reanalyzing the same data from different perspectives. The use of omics data is mostly limited to transcriptomics, copy number variations, and DNA methylations because of their abundance in different data portals. Repositories like The Cancer Genome Atlas (TCGA) (14), International Cancer Genome Consortium (ICGC) (15), Cancer Cell Line Encyclopedia (CCLE) (96), Molecular Taxonomy of Breast Cancer International Consortium (METABRIC) (97), and TARGET (98) store and share different types of transcriptomics and genomics data. TCGA contains data for 67 primary sites for more than 84,000 cases. The Clinical Proteomic Tumor Analysis Consortium (CPTAC) provides proteomics data corresponding to TCGA cohorts (99). The Personal Genome Project-United Kingdom is an open-access resource of human multi-omics data of several diseases (100). Database Gene Expression Omnibus (GEO) contains RNA and miRNA transcriptomics data (101). LinkedOmics contain data from different types of cancers (102). GliomaDB (103) and MOBCdb (104) are databases dedicated to glioma and breast cancer, respectively. The Omics Discovery Index (OmicsDI) provides a framework for accessing and disseminating omics datasets (105).

## Precision Medicine Approaches

The precision medicine initiative, launched in 2015 in United States, aims to shift from “one-size-fits-all” treatment to tailored treatment for cancer patients. To cater the need of right treatment at the right time, precision medicine uses a more individualized molecular approach and enriches pharmacogenomics (106). This individualized approach requires assembly and analysis of the individual’s molecular signatures, which could be manifested in the form of multiple types of omics data representing the status of various biomolecules for this individual. AI and other deep learning tools and techniques can be utilized to optimize the utilization of patients’ derived multi-omics data to extract target bio-entities and fit the targets with drug–target interaction data to extract relevant drugs and doses in the omics data landscape. Technologies like nanotechnology are boosting the attempt to targeted drug delivery (107). Software like G-DOC Plus provides infrastructure to explore and analyze clinical, multi-omics data at different levels, from individual to a population as a whole (108). To develop the precision medicine drugs, clinical trials also need to be reshaped with emphasis on selecting trial for patient, rather than selecting patients for trial (109, 110).

## Future Direction and Challenges

Due to the technological advances, collecting “omics” data is becoming more cost effective and will be more available. The availability of data is definitely advantageous for the analysts, as it will provide more opportunities to explore different perspectives. The use of ML in multi-omics data analysis is mostly limited to identifying the disease subtypes, biomarkers, and correlation among them. Multi-omics analysis-based disease subtype classification has shown its superiority over the conventional TNM staging method. Although the result is promising, these attempts need to be leveraged to dig out the underlying causative phenomena associated with the particular phenotype. This is definitely a challenging work because of the diversity in the data. In this review, we have discussed about the potential biomarkers already identified by several researchers for different types of cancers. However, these outcomes are still sparse in nature. It requires more studies so that this outcome can be translated to the patients. Multi-omics data analysis is still an under-developed area of research. It is a promising and fast-growing area of research. It has a lot of scope of development especially when allied data like radiomics is included. AI-driven analysis of radiomics data can overcome limitations of classical pathology. Radiomic features are promising tools in defining cancer subtypes (28, 111) and may appear as an alternative or complimenting data to primary omics data in the context of tumor classification for precision medicine (112, 113). Several other factors like lifestyle and environmental effects can be integrated to add a new dimension to the analysis. It needs a combinative effort from clinicians, biologists, and computational analysts because ML alone cannot solve the problem of causal inference (114). The primary specimens collected by healthcare persons is experimentally analyzed by the biologists and then computationally analyzed by the analysts. The extracted information is again sent back to the healthcare persons after being justified by the biologists. In such workflow, interdiscipline knowledge need to be shared in a fluent way for the fruitful outcomes for multi-omics analysis.

## Conclusion

Starting from Percivall Pott’s observation in 1775 on occurrence of scrotal cancers among the chimney sweeps due to the exposure to chimney soot, cancer research has passed a long way (115). Cancer is still a huge socioeconomical burden. Researchers, around the globe, over the centuries, have haunted for the cure of cancer. Several discoveries and inventions have enriched our understanding of cancer. In this review, we have discussed the usage and the outcomes from most recent works using ML and/or AI to analyze multi-omics data of different types of cancers. The goal of applying AI is transforming data to knowledge for the benefit of mankind. AI-based technology is proficient in identifying features in varieties of data as well as relating the features at an unprecedented speed. The effectiveness of implementing AI lies in providing better accuracy and speed in precise diagnosis and hence in clinical decision-making. We have observed successful implementations of varieties of algorithms aiming toward precision oncology. Combinations of supervised and unsupervised algorithms are used. Often essential features are identified in an unsupervised manner and then classification is performed by supervised algorithms. Different types of omics data, individually, provide information on a particular type of bio-entity. AI is needed for the integrative approach to provide a holistic view to the understanding of complex diseases like cancer. Various approaches are followed for data integration including concatenation of features extracted from individual omics data. Using AI, researchers have found out subtypes within different types of cancers along with underlying pivotal genes, proteins, RNAs, and miRNAs, which appear as potential therapeutic targets. These pivotal biomarkers further correlate with biological pathways. AI is also used to predict disease prognosis and drug response. These clinically relevant achievements are needed to be more robust for being translated toward the right treatment for the right patient. Hence, it is believed that the rapidly evolving AI-based medical data analysis is going to aid significantly the treatments in cancer.

## Author Contributions

Both the authors prepared the manuscript. Both authors contributed to the article and approved the submitted version.

## Conflict of Interest

The authors declare that the research was conducted in the absence of any commercial or financial relationships that could be construed as a potential conflict of interest.
